# Local systems, local solutions: which factors drive essential medicine availability in public health facilities across Indonesia?

**DOI:** 10.1136/bmjgh-2025-019616

**Published:** 2026-02-06

**Authors:** Relmbuss Biljers Fanda, Ari Probandari, Yuyun Yuniar, Margo van Gurp, Wouter Guus van der Hoeven, Harimat Hendarwan, Laksono Trisnantoro, Maarten Olivier Kok

**Affiliations:** 1 Erasmus University Rotterdam, Rotterdam, Netherlands; 2 Center for Health Policy and Management, Gadjah Mada University, Yogyakarta, Special Region of Yogyakarta, Indonesia; 3 Department of Public Health, Faculty of Medicine, Universitas Sebelas Maret, Surakarta, Central Java, Indonesia; 4 Research Center for Preclinical and Clinical Medicine, National Research and Innovation Agency Republic of Indonesia, Bogor, West Java, Indonesia; 5 School of Social Sciences, University of New South Wales, Sydney, New South Wales, Australia; 6 KIT Royal Tropical Institute, Amsterdam, Netherlands; 7 Amsterdam Public Health, Vrije Universiteit Amsterdam, Amsterdam, Netherlands; 8 Universitas Indonesia Maju, Jakarta, Indonesia; 9 Department of Health Policy and Management, Gadjah Mada University, Yogyakarta, Special Region of Yogyakarta, Indonesia

**Keywords:** Health systems evaluation, Public Health, Health Services Accessibility, Interdisciplinary Research

## Abstract

**Introduction:**

Ensuring free access to essential medicines is a cornerstone of universal health coverage, yet many countries face persistent local disparities in medicine availability. This study investigates the factors driving variation in essential medicine availability in primary health facilities across Indonesia, focusing on the functionality of Local Pharmaceutical Systems (LOPHAS) and the influence of socioeconomic and geographical environments.

**Methods:**

Enumerators visited each of the 514 district health offices and 9831 primary health centres (PHCs) to conduct a nationwide health facility assessment. These data were combined with publicly available information on spatial, geographical, socioeconomic and health system factors. Using regression analysis, multilevel modelling and spatial autocorrelation techniques, we identified facility-level, district-level and provincial-level factors associated with the availability of 50 essential medicines in public health facilities.

**Results:**

On average, 66% out of 50 surveyed medicines were available in PHCs, with district-level availability ranging from 83% in top-performing areas to just 43% in the lowest. PHCs with a pharmacist, clear guidelines and proper storage infrastructure had significantly higher availability, compared with those without. Other key drivers included the application of inventory management principles (eg, First-Expired, First-Out), autonomy in procurement and district level stock levels. Spatial analysis revealed strong clustering of medicine availability within a 2 km radius (Moran’s I: 0.67), with high-availability clusters present even in low-performing districts, highlighting the role of localised factors.

**Conclusion:**

Essential medicine availability in Indonesian PHCs varies substantially and is closely linked to the functionality of local pharmaceutical systems. Strengthening human resources—particularly by ensuring the presence of a pharmacist in every PHC—and improving physical infrastructure are critical priorities. Beyond PHC-level interventions, targeted efforts to enhance the capacity of district health offices in managing pharmaceutical supply chains are essential, especially in rural and remote districts of eastern Indonesia.

**WHAT IS ALREADY KNOWN ON THIS TOPIC**

- The Indonesian government is striving to achieve universal health coverage, which includes providing free access to essential medicine in public health facilities.
- While medicine stockouts in public health facilities are a known concern, little is understood about how in the decentralised health system in Indonesia, facility and district level factors, and geographical and socioeconomic characteristics drive variations in medicine availability.

**WHAT THIS STUDY ADDS**

- This study reveals significant variation in the availability of 50 essential medicines across districts, ranging from 83% in the best-performing areas to just 43% in the lowest-performing districts.
- Using a Local Pharmaceutical Systems (LOPHAS) approach, this study identifies multiple drivers of essential medicine availability. Key contributors include the presence of pharmacists at primary health centres (PHCs), adequate storage infrastructure, the application of inventory management principles and autonomy in medicine procurement.
- District level factors, such as comprehensive district planning, diversified procurement strategies and the availability of medicines at district storage facilities, are also critical for ensuring a consistent medicine supply to PHCs.

**HOW THIS STUDY MIGHT AFFECT RESEARCH, PRACTICE, OR POLICY**

- Identifying key local system features that significantly impact medicine availability can guide policymakers in strengthening pharmaceutical management at both PHC and district levels, particularly in rural and underserved regions.

## Introduction

 Around the world, many countries struggle to provide access to medicine to their population. Studies consistently reveal considerable disparities in medicine availability between different regions within countries.^1 2^ These local variations exacerbate inequalities, as medication availability is often higher in richer urban areas and lower in rural and remote areas, where residents typically have lower incomes and more limited access to healthcare.^3^,^6^ Limited availability of medicines, combined with high prices, poses significant risks to patients’ health by increasing the likelihood of untreated conditions, suboptimal care and inflated healthcare costs. Furthermore, the scarcity of medicines in public facilities often drives patients to seek alternatives from private, unregulated sources, which raises the risk of exposure to expensive, expired or falsified medications.^7^,^11^

Previous studies indicate that several factors influence the availability of essential medicines. These include infrastructural, geographical, socioeconomic aspects, as well as the organisation of the health and pharmaceutical systems.^12 13^ Poor road conditions, limited transportation options and long travel times can significantly hinder access to medicines. Additionally, geographical and political challenges—such as rurality, distance from distribution points, poor governance and insecurity—are well-documented barriers to maintaining a consistent supply of essential medicines.^14^ In contrast, effective procurement, proper storage and dispensing practices, well-trained personnel, real-time monitoring, transparent funding mechanisms and a strong health and pharmaceutical system significantly enhance access to medicines.^15 16^

One of the countries striving to improve access to essential medicine in public facilities is Indonesia, the world’s fourth most populus nation. Indonesia faces a unique set of challenges. The geographic, social and economic diversity of this vast archipelago country is exceptional.^17^ More than half of its population of 270 million people are squeezed into Java Island, just the 6% of the land mass.^18^ The other 120 million citizens are scattered across some other 7000 islands, spread out over the area of 5.1 million square kilometres.^19^ While fiscal capacity of districts in Java tends to be classified as high and very high, the others, especially rural districts in Eastern Indonesia, were mostly classified as low and very low.^20^

Indonesia has managed to sign up 98% of its population for its mandatory national health insurance scheme, which promises free access to essential medicines in public facilities.^17 21^ Primary health centres (PHCs) play a key role in providing health services and ensuring access to medicines. There are over 10 000 PHCs, which are strategically located throughout the archipelago and are the most accessible health service points for the population.^22 23^ A recent study indicates that while the availability of the 17 most-needed essential medicines in PHCs is relatively high at 82%, the availability drops to 58% when considering a broader selection of 60 essential medicines.^18 24^

A major concern is the significant variation in medicine availability across the country. Medicine stockouts are most frequently reported in more remote and peripheral areas in Indonesia, especially in the rural districts in the east, where the population tends to have the lowest income and highest health need, and access to complementary medicine dispensing points, such as hospitals and drug stores, is also most limited.^25^

To reduce the stark local variation and improve access to medicines across Indonesia, analysts should not only focus on national policies and regulations but also examine the local factors that shape medicine availability. Indonesia has 514 district health offices (DHOs), which play a key role in allocating staff, such as pharmacists, and in managing the supply of medicines to (PHCs).^26 27^ DHOs procure, store and distribute medicines to PHCs within their districts. Large PHCs classified as financially independent are permitted to procure their own medicines, while other PHCs may do so only in emergency situations and with DHO approval.

Several aspects of DHOs and PHCs can influence their capacity to manage pharmaceutical supplies, including the availability of trained personnel, adequate facilities, transportation capacity, sufficient funding and effective monitoring systems. A core idea underpinning this study is that these interrelated local factors collectively form and function as a Local Pharmaceutical System (LOPHAS), which is inspired by the existing conceptual framework of a pharmaceutical system.^1 2^ ^28^ A local pharmaceutical system comprises the people, structures, resources, practices and interactions at the subnational level that contribute to ensuring access to medicines, promote their appropriate use and support the delivery of health services that improve health outcomes.^28^ In Indonesia, these local systems encompass community-level, health facility-level and district-level entities that operate within the broader national health and pharmaceutical system.

Local circumstances, including health needs, the socioeconomic situation and logistical and geographical factors, are also related to the local availability of medicines. Local differences in health needs, health insurance coverage, income levels and poverty rates directly influence the demand for medicines. Logistical factors also differ for different areas, including the travel time, infrastructure quality and the need to cross the sea to travel to another island.^29^

Although multiple factors influence the performance of local pharmaceutical systems, it remains unclear which are most critical for ensuring access to medicines. Generating new insights into the key drivers of medicine availability is essential for guiding interventions, targeting investments, improving access, reducing inequalities and advancing understanding of effective strategies to achieve universal access to medicines in a large, diverse and decentralised nation.

In this study, we assess the availability of essential medicine in PHCs in Indonesia and analyse the local pharmaceutical, health system, socioeconomic and geographical indicators driving variation in medicines availability. In 2019, enumerators visited each of the 514 DHOs and 9831 PHC to conduct a health facility assessment and collect data on the availability of essential medicines. We analysed the availability of 50 essential medicines and explored its relationship with the organisation of the local pharmaceutical system, health insurance coverage, district fiscal capacity, health expenditure and demographic and geographical indicators, such as the distance to the district capital. Since the PHCs are nested within districts and provinces, we conducted a multilevel analysis. To gain insight into the geographical clustering of medicines availability, we also performed a spatial autocorrelation analysis.

## Methods

This article presents the results of a nationwide health facility survey, for which enumerators visited all 514 district health offices and all primary health centres (Pusat Kesehatan Masyarakat) across Indonesia. The 2019 cross-sectional survey, named Riset Fasilitas Kesehatan (Rifaskes), was organised by the Indonesian National Institute of Health Research and Development (Balitbangkes), with technical guidance and support from academic experts. For our analysis, we combined this with additional data regarding district characteristics related to accessibility, financial and demographical determinants and geographical and spatial information, which were derived from four other datasets. These are (1) the fiscal capacity map of Indonesian provinces and districts, (2) local government expenditures data containing provinces and districts, (3) health insurance coverage per district and (4) village potential survey.^30 31^ A detailed description of the Local Pharmaceutical Systems (LOPHAS) approach and framework can be found in online supplemental figure 1 and online at: https://dataverse.harvard.edu/dataverse/Local_Pharmaceutical_System.

### Sample size and data collection

The data in the Rifaskes study were gathered on site from all 514 DHOs and 9909 PHCs in Indonesia. We included all registered PHCs that were listed as active at the start of 2019 and were functioning as PHCs when physically reached by the enumerators (n=9831 PHCs).

### Data collection and management

The data collection began with a pilot phase in Cirebon District, West Java. The pilot phase was led by staff of the Ministry of Health, who later participated as field coordinators at the provincial level.

Data collectors interviewed the PHC staff with a paper-based structured questionnaire and submitted the data through the Redcap online platform. To control the quality of data collection, the field coordinators provided daily supervision and regularly checked the performance of each enumerator. Field coordinators checked the data and ensured that data in the system matched the paper questionnaire. The electronic data were stored at the data management unit at the National Institute for Health Research and Development. Data were ready to be used in 2021 after compiling them in 2020. More details about the research method have been published in the Rifaskes report.^32^

The additional datasets were derived from two ministry data repositories and the Indonesian statistics agency. The data about PHCs’ characteristics were derived from the Ministry of Health, and data about fiscal capacity of districts and provinces and local government expenditure were obtained from the Ministry of Finance.

Spatial data concerning the euclidean distance (km) from each PHC to the district capital, provincial capital and nearest PHC were calculated using QGIS software based on latitude and longitude coordinates.

All data were merged before being statistically analysed using district and province codes provided by the Ministry of Home Affairs. We had 36 observations with missing values. These cases were excluded from the analyses through listwise deletion. Given the small number of missing observations, we believe that their exclusion did not have a substantial effect on our results.

### Variables

Our main outcome variable was the availability of 50 essential medicine availability at DHOs and PHCs. The selection of these 50 medicines was guided by the WHO’s Service Availability and Readiness Assessment (SARA) instrument and finalised in consultation with the Indonesian Ministry of Health. Medicines that are only relevant in specific regions, such as antimalarials, were excluded to ensure national applicability. Enumerators visited PHCs and DHOs and physically verified the presence of each of the 50 selected essential medicines at the facility. A medicine was considered available if a full course for at least one patient was present in the facility’s pharmacy at the time of the visit. The outcome variable was then calculated as the percentage of the 50 essential medicines that were available at each facility. In alignment with Indonesia’s national medicine policy, two pairs of medicines were treated as therapeutically substitutable: glibenclamide and metformin (oral antidiabetic agents), and furosemide and hydrochlorothiazide (HCT) (antihypertensive/diuretic agents). For each pair, the presence of at least one of the two medicines was considered sufficient to indicate availability within the respective therapeutic category.

To determine which variables are associated with medicine availability, we used the local pharmaceutical system approach and adapted it to the Indonesian context. We focused our analysis on four core system components (1) managing human and physical resources, (2) financing, (3) monitoring performance and (4) managing pharmaceutical supply. We evaluated the functioning of these core components and their impact on the availability of medicines in PHCs. Our analysis also considered other external factors, including demographic, social and geographic characteristics.^33^ Online supplemental table 1 shows all covariates and their operational definitions.

### Statistical analyses

**Figure 1:**
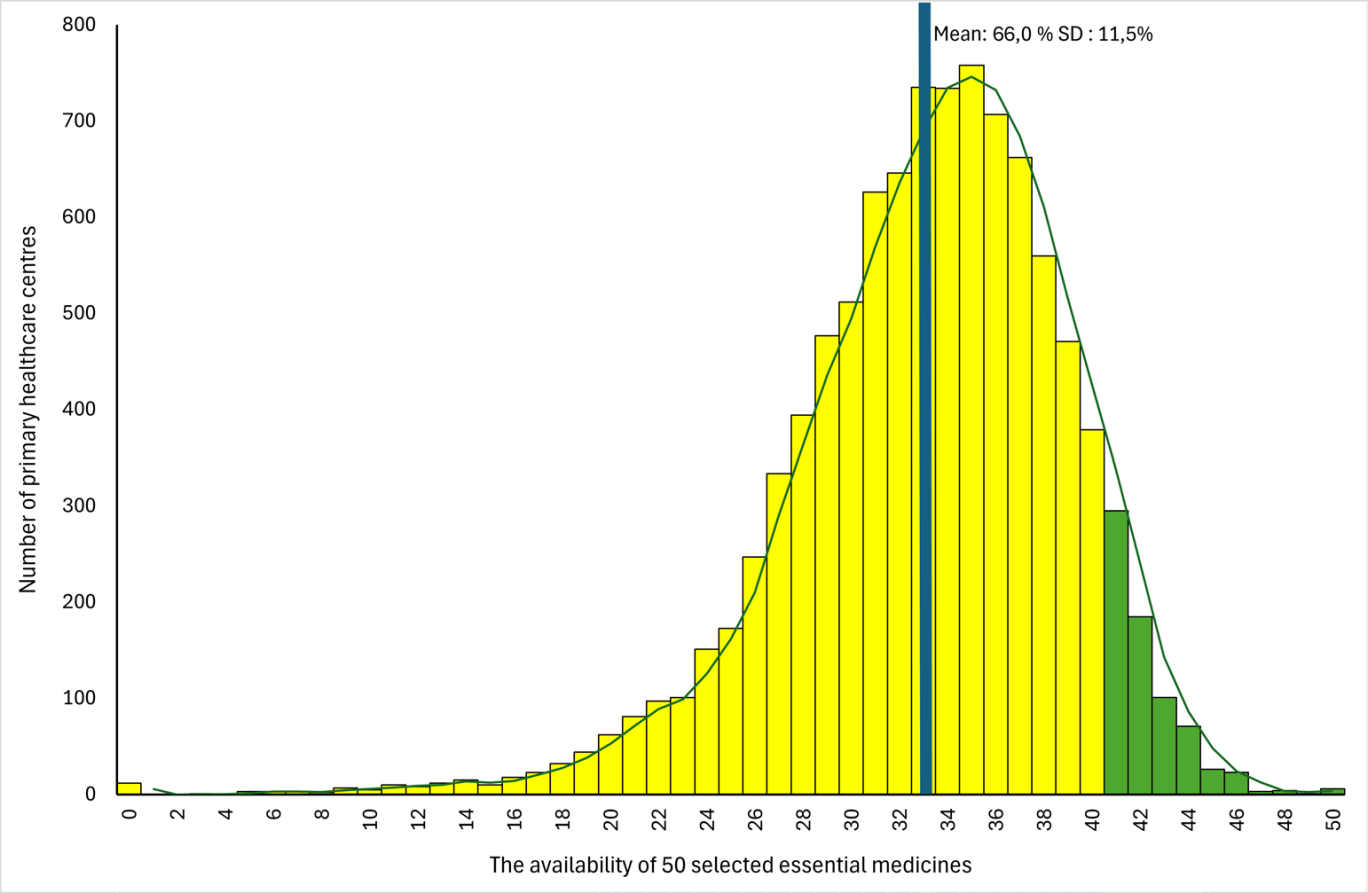
Distribution of the medicine availability in primary health centres in Indonesia in 2019.

We started the analysis by inspecting the outcome variable. The medicine availability variable was normally distributed (c.f. figure 1). For each covariate, we calculated the mean and SD of the outcome. For numerical covariates, we presented the median and IQRs. We continued our statistical analysis in three steps.

The first step is selecting covariates based on the multivariable analyses within subdimensions. Covariates were tested for multicollinearity prior to model building, covariates with a significant correlation coefficient beyond 0.8 were excluded. Next, separate multivariable models were built for each subdimension. Using a backward selection process, we removed variables from the model that were not statistically significant at the 5% level. The remaining variables were included in the full models. We presented the univariable and multivariable results (crude model).

In the second step, we considered two different methods to account for the dependency of medicine availability between PHCs: (1) by fitting a multilevel model with a random intercept on province and district level, and (2) by fitting a spatial lag model using the average medicine availability of two nearest neighbouring PHCs. We first investigated the strength and scale of spatial autocorrelation in medicine availability by computing the Moran’s I statistic at various district bands. We then tested the residuals of the multilevel model and the spatial-lag model for spatial autocorrelation to ensure that the models accounted for the spatial dependency in medicine availability. We compared the fit of the crude model, the multilevel model and the spatial lag model based on the Akaike information criterion (AIC) and R-squared (R^2^). The residuals and predictions of both models were visualised to ensure assumptions of linear regression analyses hold (online supplemental figure 2).

In the final step, we assessed the contribution of each subdimension of the LOPHAS framework to the multilevel and spatial models. This was done by removing all variables from a single subdimension (eg, financing) from the models and evaluating the change in R^2^ and AIC.

The descriptive and regression analyses were performed using Stata version 18. The assessment of spatial autocorrelation was performed using GeoDa version 1.12.

## Patient and Public Involvement

Patients or the public were not involved in the design, or conduct, or reporting, or dissemination plans of our research.

## Results

We start with our descriptive analysis. Next, we present the analysis of spatial autocorrelation. We then present the statistical results of multilevel and spatial lag models. Our investigations of collinearity and multivariable analyses within subdimension result in 50 covariates that can be used for the statistical analyses.

### Descriptive analyses

On average, 66% of the 50 essential medicines were available in the PHCs as presented in figure 1. Table 1 illustrates the availability of medicines in PHCs, taking into account 58 covariates with nominal and ordinal scales. The numerical covariate descriptions can be found in online supplemental table 2. They represent the characteristics of PHCs, DHOs and district contexts. The result of the multivariable analysis can be found in online supplemental material 2.

**Table 1:** The average of medicine availability based on geographical accessibility and fiscal capacity, district health office and primary health centre characteristics

	The availability of 50 medicines			
	**N**	**%**	**Mean**	**SD**
PHC level				
Output				
Medicine availability	9831	100.00	66.00	11.50
Local pharmaceutical systems				
Managing human and physical resources				
The availability of pharmacists	7630	77.61	67.17	10.58
The availability of medicine shelves	9483	96.46	66.42	11.11
Perceived of a sufficient number of shelves	5886	59.87	66.92	11.01
The availability of lighting	9066	92.22	66.51	11.14
The availability of the guidelines of medicine and medical product services	8393	85.37	66.96	10.91
The availability of the guidelines for preparing and dispensing medicine concoction prescription	8702	88.52	66.91	10.89
Inpatient services	4094	41.64	68.70	11.06
Accredited	7569	76.99	67.71	10.5
The availability of ventilation or air circulation	8628	87.76	66.44	11.19
The availability of a separate room for pharmaceutical products	9548	97.12	66.38	11.15
The availability of the guidelines for preparing and dispensing dry syrup	7848	79.83	66.99	10.99
Financing				
Receiving district government fund	6289	63.97	66.73	11.08
Receiving retribution from patients	6569	66.82	67.75	10.6
Receiving a non-capitation fund from the National Health Insurance Agency (BPJS Kesehatan)	6570	66.83	67.87	10.5
Receiving a central government fund	7562	76.92	66.11	11.57
Receiving the capitation fund from the National Health Insurance Agency (BPJS Kesehatan)	9441	96.03	66.26	11.44
Having independent financial autonomy	3239	32.95	68.24	10.62
Monitoring performance				
Having the PHC management information system (SIMPUS)	6087	61.92	66.82	11.22
Having an electronic PHC management information system (SIMPUS)	4257	43.30	67.78	10.66
Having an online PHC management information system (SIMPUS)	3430	34.89	67.98	10.47
Managing pharmaceutical product supply				
Developing an independent medicine quantification	8119	82.59	66.81	10.81
The availability of medicine use and ordering a monthly report (LPLPO)	9722	98.89	66.17	11.44
The completeness of LPLPO	9351	95.12	66.41	11.22
Implementing DRP planning for an 18-month period	7285	74.10	66.76	11.22
Sources of medicine—combining DHO & PHC	3984	40.52	67.92	10.5
Achieving the volume target	6383	64.93	66.72	11.1
Procuring using the capitation fund via e-purchasing scheme	3612	36.74	67.92	10.61
Procuring using the capitation fund through direct purchasing	5229	53.19	67.21	10.88
Note for medicine in and out in 2018	9343	95.04	66.56	11.02
Apply FIFO and FEFO methods	9036	91.91	66.86	10.77
Reporting rational use of medicines	8430	85.75	67.10	10.72
Sources of medicine—only DHO procurements	5665	57.62	64.85	12.09
Able to use the capitation fund	8437	85.82	66.45	11.38
Independent procurement of medicines using a capitation fund	3625	36.87	66.74	11.22
Make independent medicine needs plan (Rencana Kebutuhan Obat)	9237	93.96	66.46	11.17
All medicines were from the PHC procurement	150	1.53	64.23	11.89
Accessibility, financial and demographical determinants				
PHC type				
Remote/very remote (reference)	2192	22.30	62.57	13.75
Rural	4663	47.43	67.15	11.08
Urban	2976	30.27	67.02	9.86
District level				
Local pharmaceutical systems				
Managing human and physical resources				
Having a pharmacist as the PIC at district warehouses	8082	82.21	66.29	11.53
Availability of pharmacist staff	8864	90.16	66.08	11.52
Having a staff with a pharmacy background as the PIC at the district warehouses	8568	87.15	66.22	11.58
Financing				
Financial resources for the medicine procurements—national fund	9162	93.19	66.22	11.6
Financial resources for the medicine procurements—provincial fund	1090	11.09	69.2	11.47
Financial resources for the medicine procurements—local fund	4149	42.2	67.31	11.11
Financial resources for the medicine procurements—capitation	2515	25.58	66.4	12.29
Managing pharmaceutical product supply				
Conduct medicine quantification	9550	97.14	66.09	11.63
Procurement schemes—national e-catalogue platform	9241	94.00	66.29	11.43
Procurement schemes—local auction	4337	44.12	66.75	11.77
Procurement schemes—local direct purchasing	4635	47.15	67.38	11.28
Implement procurement medicine policy: over 2 years of date expiration	9316	94.76	66.15	11.51
All medicines procured with policy: over 2 years of date expiration	6416	65.26	65.83	11.74
Waiting time for medicine procurement is less than 1 month	2356	23.97	67.12	10.95
Accessibility, financial and demographical determinants				
District type				
Rural	8131	82.71	66.08	11.93
Urban	1700	17.29	66.14	9.54
Percentile of the total district expenditure				
Quartile 1	1292	13.14	62.85	12.14
Quartile 2	1324	13.47	64.15	12.19
Quartile 3	1688	17.17	64.44	12.22
Quartile 4	2144	21.81	68.23	11.36
Quartile 5	3383	34.41	67.55	10.25
Percentile of district population				
Quartile 1	1127	11.46	60.73	14.31
Quartile 2	1241	12.62	64.62	12.03
Quartile 3	1731	17.61	65.30	11.73
Quartile 4	2136	21.73	67.06	10.97
Quartile 5	3596	36.58	68.08	9.92
District fiscal capacity				
Low and very low	3535	35.96	63.66	12.48
Medium	2315	23.55	67.79	11.52
High and very high	3981	40.49	67.26	10.28
Located on a separate island from its provincial capital	1267	12.89	63.41	13.37
Provincial level				
Accessibility, financial and demographical determinants				
Region				
Eastern Indonesia	1215	12.36	60.12	14.59
Sumatera	2547	25.91	65.06	11.48
Borneo, West Nusa Tenggara, Sulawesi	2373	24.14	65.89	11.04
Java and Bali	3696	37.60	68.89	9.77
Provincial fiscal capacity				
Low and very low	2661	27.07	64.57	12.2
Medium	2299	23.39	66.53	10.53
High and very high	4871	49.55	66.71	11.57

DHO, district health office; DRP, drug requirement plan; FEFO, First-Expired, First-out; FIFO, First-In, First-out; LPLPO, Laporan Pemakaian dan Lembar Permintaan Obat; PHC, primary health centre.

### Spatial Autocorrelation

**Figure 2:**
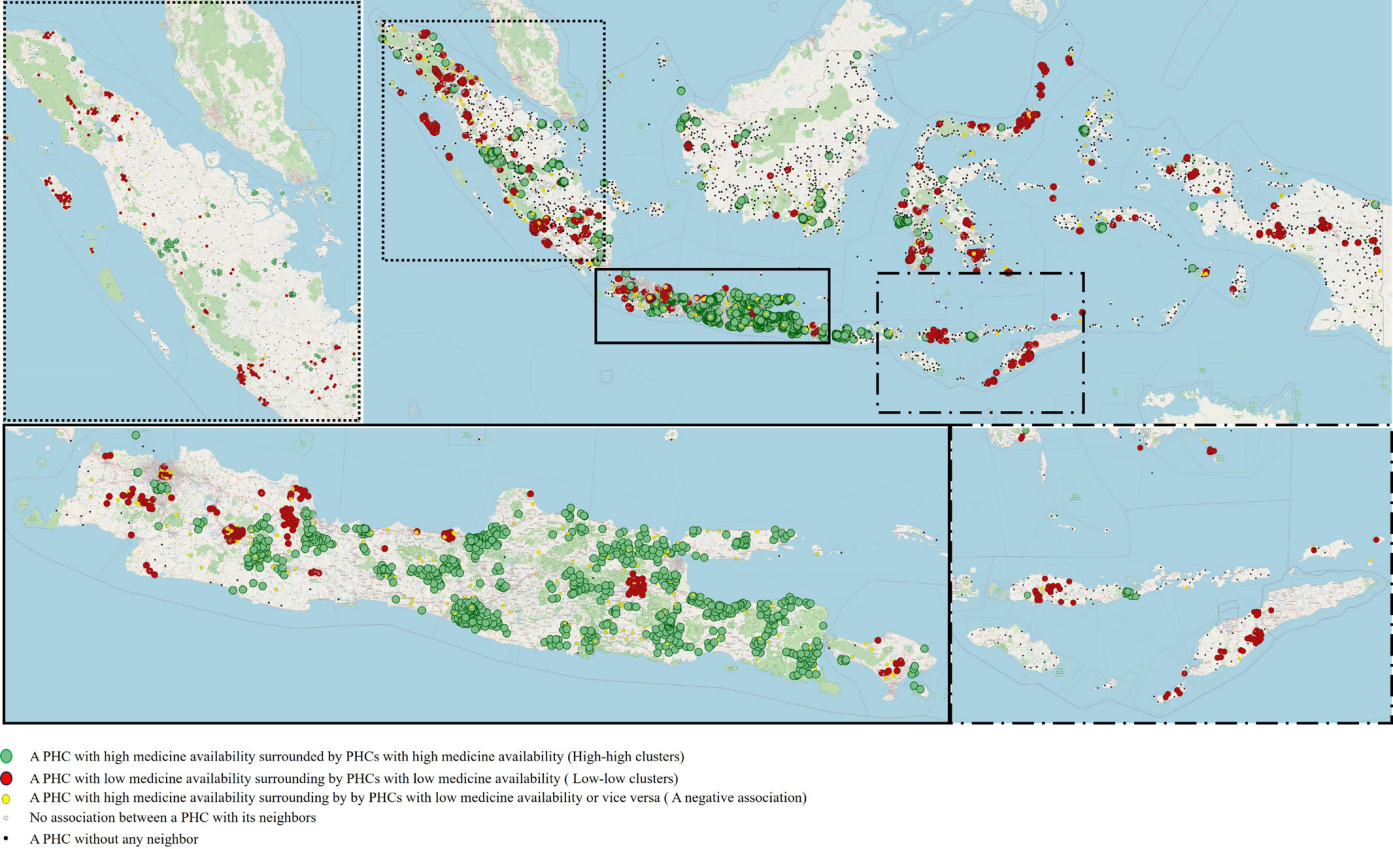
The map of a hotspot analysis to identify clusters of medicine availability at the PHC level in Indonesia. PHC, primary health centre.

The medicine availability of a PHC tends to be similar to its neighbours. Online supplemental figure 3 presents Moran’s I statistic across various distance bands and shows Moran’s I value of 0.34 and p value of <0.05, indicating that the availability of medicines is spatially clustered. Spatial autocorrelation was highest for PHCs within 2 km of one another (Moran’s I: 0.67), indicating that PHCs in close vicinity of one another have similar levels of medicine availability as presented in figure 2 and online supplemental figure 4.

### Regression models

The multilevel and spatial lag models are presented in table 2. These models provide a better fit compared with the crude model based on the AIC and R2. They also account for all spatial autocorrelation.

**Table 2:** Univariable, multilevel and spatial autocorrelation analyses of the association between the availability of 50 essential medicines and its determinants at the PHC, district and provincial levels

	Univariable		Multilevel		Spatial autocorrelation	
	B	CI	B	CI	B	CI
PHC level						
Local pharmaceutical systems						
Managing human and physical resources						
The availability of pharmacists	2.43***	(2.16 to 2.70)	0.92***	**(0.67 to 1.16**)	1.06***	**(0.82 to 1.31**)
The availability of medicine shelves	1.03***	(0.80 to 1.26)	0.31	(−0.44 to 1.06)	−0.10	(−0.90 to 0.70)
Perceived of a sufficient number of shelves	4.75***	(4.14 to 5.36)	−0.16	(−0.36 to 0.04)	−0.15	(−0.36 to 0.06)
The availability of lighting	2.74***	(2.32 to 3.16)	0.46*	**(0.05 to 0.87**)	0.41	(−0.02 to 0.85)
The availability of the guidelines of medicine and medical product services	2.99***	(2.67 to 3.31)	0.50**	**(0.14 to 0.85**)	0.42*	**(0.04 to 0.80**)
The availability of the guidelines for preparing and dispensing medicine concoction prescription	3.59***	(3.24 to 3.95)	0.45*	**(0.04 to 0.85**)	0.47*	**(0.04 to 0.90**)
Inpatient services	2.24***	(2.01 to 2.46)	1.28***	**(1.07 to 1.49**)	1.47***	**(1.25 to 1.68**)
Accredited	3.52***	(3.26 to 3.78)	0.74***	**(0.47 to 1.01**)	0.78***	**(0.51 to 1.06**)
Financing						
Receiving district government fund	1.61***	(1.37 to 1.85)	−0.01	(−0.27 to 0.25)	−0.08	(−0.29 to 0.14)
Receiving retribution from patients	0.89***	(0.65 to 1.13)	0.38**	**(0.10 to 0.67**)	0.34**	**(0.10 to 0.59**)
Receiving a non-capitation fund from the National Health Insurance Agency	2.50***	(2.26 to 2.74)	0.33*	**(0.07 to 0.59**)	0.27*	**(0.02 to 0.52**)
Having Independent financial autonomy	2.68***	(2.45 to 2.92)	0.02	(−0.33 to 0.36)	0.20	(−0.03 to 0.44)
Monitoring performance						
Having an online Puskesmas Management Information System (SIMPUS)	1.46***	(1.22 to 1.69)	0.20	(−0.02 to 0.43)	0.10	(−0.13 to 0.32)
Managing pharmaceutical product supply						
Implementing Back-Referral Programme (PRB)	2.08***	(1.78 to 2.38)	0.54***	**(0.28 to 0.80**)	0.45***	**(0.18 to 0.71**)
The availability of medicine use and ordering a monthly report (LPLPO)	3.88***	(2.79 to 4.96)	1.76***	**(0.78 to 2.74**)	1.54**	**(0.50 to 2.58**)
The completeness of LPLPO	3.28***	(2.75 to 3.80)	0.26	(−0.24 to 0.76)	0.36	(−0.16 to 0.88)
Implementing DRP planning for an 18-month period	1.29***	(1.03 to 1.55)	0.39***	**(0.16 to 0.62**)	0.20	(−0.04 to 0.43)
Sources of medicine—combining DHO & PHC procurements	1.54***	(1.31 to 1.77)	0.51***	**(0.28 to 0.74**)	0.28*	**(0.06 to 0.51**)
Achieving the volume target	0.90***	(0.66 to 1.14)	0.05	(−0.16 to 0.25)	0.26*	**(0.05 to 0.46**)
Procuring using capitation fund via the e-purchasing scheme	1.45***	(1.21 to 1.68)	0.31*	**(0.06 to 0.55**)	0.31**	**(0.08 to 0.53**)
Procuring using capitation fund through a direct purchasing	1.20***	(0.97 to 1.42)	0.58***	**(0.36 to 0.80**)	0.49***	**(0.27 to 0.70**)
Note for medicine in and out in 2018	4.73***	(4.21 to 5.24)	0.23	(−0.39 to 0.84)	0.47	(−0.18 to 1.12)
Apply FIFO and FEFO methods	4.74***	(4.33 to 5.15)	1.14***	**(0.71 to 1.58**)	1.68***	**(1.22 to 2.14**)
Reporting rational use of medicines	3.56***	(3.24 to 3.88)	0.92***	**(0.60 to 1.23**)	0.94***	**(0.63 to 1.25**)
Accessibility, financial and demographical determinants						
PHC type						
Remote/very remote						
Rural	2.29***	(2.00 to 2.58)	0.27	(−0.04 to 0.58)	0.07	(−0.24 to 0.37)
Urban	2.23***	(1.91 to 2.54)	0.53**	**(0.16 to 0.91**)	0.26	(−0.12 to 0.63)
Distance between PHC and its district capital point	−0.01***	(−0.01 to −0.00)	0.00	(−0.00 to 0.00)	0.00	(−0.00 to 0.00)
Distance between PHC and the nearest PHC	−0.00	(−0.00 to 0.00)	−0.00	(−0.00 to 0.00)	−0.00	(−0.00 to 0.00)
District level						
Local pharmaceutical systems						
Managing human and physical resources** **						
Having a pharmacist as the PIC at district warehouses	0.58***	(0.28 to 0.88)	0.12	(−0.47 to 0.70)	−0.12	(−0.39 to 0.14)
Financing						
Financial resources for the medicine procurements—national fund	0.96***	(0.50 to 1.41)	0.23	(−1.00 to 1.45)	0.14	(−0.42 to 0.69)
Financial resources for the medicine procurements—provincial fund	1.75***	(1.39 to 2.11)	0.78*	**(0.00 to 1.56**)	0.72***	**(0.40 to 1.05**)
Financial resources for the medicine procurements—local fund	1.06***	(0.83 to 1.29)	−0.34	(−0.86 to 0.18)	−0.15	(−0.37 to 0.08)
Managing pharmaceutical product supply						
Conduct medicine quantification	0.03	(−0.65 to 0.72)	−4.06**	**(−6.61 to −1.51**)	−2.56***	**(−3.44 to −1.68**)
Procurement schemes—e-catalogue	1.68***	(1.20 to 2.16)	−1.05	(−2.41 to 0.31)	−1.93***	**(−2.56 to −1.30**)
Procurement schemes—local auction	0.59***	(0.36 to 0.82)	0.65*	**(0.13 to 1.16**)	0.27*	**(0.04 to 0.49**)
Procurement schemes—local direct purchasing	1.23***	(1.00 to 1.45)	0.48	(−0.03 to 0.98)	0.33**	**(0.11 to 0.55**)
Implement procurement medicine policy: over 2 years of date expiration	0.63*	(0.12 to 1.14)	0.73	(−0.36 to 1.81)	0.36	(−0.13 to 0.84)
All medicines procured with policy: over 2 years of date expiration	−0.37**	(−0.61 to −0.13)	−0.16	(−0.68 to 0.37)	−0.04	(−0.26 to 0.19)
Waiting time for medicines is less than 1 month	0.68***	(0.41 to 0.94)	0.03	(−0.57 to 0.62)	0.23	(−0.03 to 0.49)
Medicine availability at the DHO level						
The availability of 50 medicines	0.15***	(0.13 to 0.16)	0.20***	**(0.17 to 0.24**)	0.14***	**(0.12 to 0.16**)
Accessibility, financial and demographical determinants						
District type						
Rural						
Urban	0.03	(−0.27 to 0.33)	−0.65	(−1.45 to 0.15)	−0.52*	**(−0.92 to −0.12**)
Percentile of the total district expenditure						
Quartile 1						
Quartile 2	0.65**	(0.21 to 1.08)	0.63	(−0.17 to 1.42)	0.27	(−0.14 to 0.68)
Quartile 3	0.79***	(0.38 to 1.21)	0.23	(−0.61 to 1.08)	0.05	(−0.36 to 0.46)
Quartile 4	2.69***	(2.30 to 3.08)	0.47	(−0.46 to 1.40)	0.33	(−0.11 to 0.77)
Quartile 5	2.35***	(1.99 to 2.72)	−0.93	(−2.09 to 0.23)	−0.76**	**(−1.27 to −0.26**)
Percentage of subsidised JKN insurance participants	−0.03***	(-0.03 to −0.02)	−0.01	(−0.03 to 0.01)	−0.01*	**(−0.02 to −0.00**)
District fiscal capacity						
Low and very low						
Medium	2.06***	(1.76 to 2.36)	0.42	(−0.25 to 1.10)	0.45**	**(0.14 to 0.76**)
High and very high	1.80***	(1.54 to 2.06)	0.69	(−0.19 to 1.57)	0.68***	**(0.30 to 1.06**)
Separated from the provincial capital	−1.54***	(−1.88 to −1.20)	0.01	(−0.00 to 0.03)	−0.00	(−0.01 to 0.01)
Accessibility score to other dispensing points	0.06***	(0.05 to 0.06)	0.20	(−0.60 to 1.00)	−0.29	(−0.64 to 0.07)
Provincial level						
Accessibility, financial and demographical determinants						
Region						
Eastern Indonesia						
Sumatera	2.47***	(2.09 to 2.85)	0.90	(-0.52 to 2.31)	0.28	(−0.20 to 0.77)
Borneo, West Nusa Tenggara, Sulawesi	2.88***	(2.50 to 3.27)	0.79	(−0.60 to 2.19)	0.05	(−0.41 to 0.50)
Java and Bali	4.39***	(4.02 to 4.75)	1.47	(−0.19 to 3.13)	0.67*	**(0.08 to 1.26**)
Percentage of health expenditure from total expenditure	0.19***	(0.16 to 0.22)	−0.01	(−0.13 to 0.11)	0.00	(−0.03 to 0.03)
Provincial fiscal capacity						
Low and very low						
Medium	0.98***	(0.66 to 1.30)	−0.07	(−1.05 to 0.91)	−0.23	(−0.53 to 0.07)
High and very high	1.07***	(0.80 to 1.34)	0.19	(−0.86 to 1.23)	−0.12	(−0.43 to 0.20)
Spatial lag					0.00***	(0.00 to 0.00)

*p<0.05, **p<0.01, **p<0.001.

In the multilevel and spatial autocorrelation model, we have marked the significant variables in bold.

DHO, district health office; DRP, drug requirement plan; FEFO, First-Expired, First-out; FIFO, First-In, First-out; JKN, Jaminan Kesehatan Nasional; LPLPO, Laporan Pemakaian dan Lembar Permintaan Obat; PHC, primary health centre; PRB, Program Rujuk Balik.

At the PHC level, both models provide similar results. There are 16 variables which matter to the availability of medicines at the PHC level in three subdimensions of the local pharmaceutical system. For example, increased capacity for managing human and physical resources (eg, with pharmacists, lighting, guidelines, inpatient service and being accredited) was associated with a higher availability of essential medicines (coefficients range from 0.42 to 1.47). In addition, better managing the medicine supply chain (eg, with the availability of the monthly medicine reports, ability to use capitation fund and medicine management techniques) was also positively associated with medicine availability. Furthermore, in the multilevel model, urban PHCs were associated with a slightly higher medicine availability as compared with remote PHCs, but this association was not significant in the spatial lag model.

At the district level, various covariates describing the management of the supply chain are associated with medicine availability in PHCs, such as procuring medicines via a local auction, which had a positive coefficient. In addition, a higher availability of medicines at the DHO level is also associated with a higher medicine availability at the PHC level.

The main difference between the two models is that covariates such as the type of district, the percentage of subsidised National Health Insurance participants and fiscal capacity are associated with medicine availability in the spatial lag model, but not in the multilevel model.

At the provincial level, only the region variable was associated with medicine availability at the PHC level. There was an 8.8% disparity in medicine availability between PHCs in Eastern Indonesia and those on the Java-Bali region in the descriptive table. The spatial autocorrelation analysis affirms this difference with B: 0.67, CI 0.08 to 1.26.

### The contribution of the subdimensions

Table 3 shows the contribution of each subdimension and in each system tier to the multilevel and spatial lag models. Variation in medicine availability is explained most by variation in PHC level covariates, followed by district and then provincial level.

**Table 3:** The investigation of the individual dimension’s contribution in the multilevel and spatial autocorrelation models by inspections their estimator of prediction error (AIC) and coefficient of determination (R2)

	AIC change in multilevel				R2 change in spatial autocorrelation			
	Dimension only		Excl. dimension		Dimension only		Excl. dimension	
	Actual value	The changes	Actual value	The changes	Actual value	The changes	Actual value	The changes
Level of data
Full model: all dimensions	57 922	29.5%
Primary health centres level	58 024	+102 (+0.18%)	58 847	+925 (+1.6%)	26.3%	−3 (−10.85%)	22.7%	−7 (−23.05%)
District level	58 843	+921 (+1.59%)	58 031	+109 (+0.19%)	22.3%	−7 (−24.41%)	26.4%	−3 (−10.51%)
Provincial level	58 964	+1042 (+1.8%)	57 914	+−8 (−0.01%)	19.0%	−11 (−35.59%)	24.5%	−5 (−16.95%)
Local Pharmaceutical System	58 016	+94 (+0.16%)	58 919	+997 (+1.72%)	29.1%	0 (−1.36%)	20.1%	−9 (−31.86%)
Primary health centres	58 025	+103 (+0.18%)	58 814	+892 (+1.54%)	26.3%	−3 (−10.85%)	22.8%	−7 (−22.71%)
Managing physical and human resources	58 422	+500 (+0.86%)	58 229	+307 (+0.53%)	24.2%	−5 (−17.97%)	26.8%	−3 (−9.15%)
Financing	58 877	+955 (+1.65%)	57 942	+20 (+0.03%)	20.0%	−10 (−32.2%)	29.4%	0.1 (−0.34%)
Monitoring and evaluation	58 963	+1041 (+1.8%)	57 923	+1 (+0%)	17.7%	−12 (−40%)	29.5%	0 (0.00%)
Managing supply chain	58 401	+479 (+0.83%)	58 270	+348 (+0.6%)	22.5%	−7 (−23.73%)	28.1%	−1 (−4.75%)
District health offices	58 869	+947 (+1.63%)	58 036	+114 (+0.2%)	20.0%	−9 (−32.2%)	26.9%	−3 (−8.81%)
Managing physical and human resources	58 967	+1045 (+1.8%)	57 920	−2 (+0.00%)	17.3%	−12 (−41.36%)	28.8%	−1 (−2.37%)
Financing	58 966	+1044 (+1.8%)	57 922	+0 (+0.00%)	17.8%	−12 (−39.66%)	29.4%	0 (−0.34%)
Managing supply chain	58 958	+1036 (+1.79%)	57 937	+15 (+0.03%)	17.9%	−12 (−39.32%)	28.8%	−1 (−2.37%)
Medicine availability—DHO	58 854	+932 (+1.61%)	58 024	+102 (+0.18%)	18.8%	−11 (−36.27%)	27.5%	−2 (−6.78%)
Accessibility, financial and demographical determinants	58 919	+997 (+1.72%)	58 016	+94 (+0.16%)	20.1%	−9 (−31.86%)	29.1%	−0.4 (−1.36%)
Primary health centres level	58 929	+1007 (+1.74%)	57 922	+0 (+0.00%)	18.2%	−11 (−38.31%)	29.5%	0 (0.00%)
District level	58 946	+1024 (+1.77%)	57 921	−1 (0.00%)	19.6%	−10 (−33.56%)	29.1%	−0.4 (−1.36%)

AIC, Akaike information criterion; DHO, district health office.

Determinants of the local pharmaceutical system (with the AIC 58016 and R-square 29.1 %) explain more variations in medicine availability than the accessibility, financial and demographic determinant (with the AIC 58919 and R-square 20.1 %). Within the local pharmaceutical system, managing human and physical resources at the PHC level and medicine availability at the DHO level explain more variations compared with the other subdimensions.

## Discussion

This study investigates the factors driving variation in essential medicine availability across primary health facilities in Indonesia, with a focus on the performance of local pharmaceutical systems and the impact of socioeconomic and geographical factors. Our findings reveal substantial disparities in medicine availability between districts. On average, only 66% of the 50 essential medicines surveyed were available in PHCs, with district-level availability ranging from 83% in the best-performing areas to just 43% in the lowest-performing ones. A strong correlation emerged between the availability of medicines at DHOs and the stock levels in PHCs within their jurisdiction. PHCs that demonstrated stronger management of human and physical resources consistently reported higher medicine availability. Furthermore, effective supply chain management at the PHC level—marked by regular medicine reporting, strategic use of capitation funds and the application of inventory management techniques—was significantly associated with improved medicine availability. These results highlight the critical role of local pharmaceutical systems in ensuring equitable access to essential medicines.

The substantial variation in medicine availability across districts in Indonesia is consistent with findings from other countries. Similar disparities have been observed in India, where availability ranged from 43% in Bihar to 88% in Tamil Nadu^3^; in China, with higher availability in the Eastern region compared with the central and western regions^34^; and in Brazil, where the Southeast region had up to 14% higher availability than the North and Northeast.^35^ These local differences highlight significant regional inequalities, emphasising the need to look beyond national indicators for medicine availability and conduct local assessments of medicine access. Beyond identifying local differences, our study contributes to the understanding of factors influencing these variations by examining both the organisation of the pharmaceutical system and the local circumstances.

The system component that is most strongly related to medicine availability is the management of human and physical resources, and especially the presence of a pharmacist. PHCs with a pharmacist had a significantly higher medicine availability compared with those without. In Indonesia, approximately 20% of PHCs operate without a pharmacist, a shortage that is particularly problematic in rural and remote areas. In the absence of a pharmacist, other staff—such as assistant pharmacists or nurses—step in to manage medicines, sometimes with support from a pharmacist at another PHC. These other professionals are not trained to make decisions on dispensing medicines, which may impact the quality of pharmaceutical services.

Our findings suggest that having a pharmacist in every PHC not only improves medication dispensing practices but also enhances medicine availability. However, increasing the number of pharmacists in remote PHCs remains a significant challenge.^36^ Studies from Indonesia and other regions indicate that pharmacists are often reluctant to work in these areas due to geographical isolation, inadequate infrastructure and lower living standards.^37^ In contrast, urban settings provide better opportunities for professional growth and additional income.^38^ Addressing these challenges requires a comprehensive approach, including improving working conditions and collaborating with health and non-health sector authorities to make rural placements more appealing.^37^

The availability of essential medicines was positively associated with procurement and storage practices. PHCs with the flexibility to use their own capitation funding for medicine procurement maintained higher stock levels than those relying solely on district health office supplies. This highlights the potential benefits of granting PHCs greater autonomy in procurement. In 2023, the Indonesian government reformed its procurement policy, allowing public healthcare facilities more flexibility to source medicines directly from suppliers. Monitoring the impact of this policy on medicine availability will be crucial, as fragmented procurement may lead to higher cost. Particular attention should be given to remote areas, where fragmented demand, high distribution costs and low order volumes could drive up prices and exacerbate shortages.^21 26^

We also found that medicine availability is positively associated with the use of inventory management principles, such as First-Expired, First-out (FEFO) and First-In, First-out (FIFO). In Indonesia’s pharmaceutical supply chain, FEFO is the preferred method for managing medicine stocks, as it helps to ensure that expired products do not reach patients. Its implementation is seen as critical for minimising waste, ensuring patient safety and maintaining regulatory compliance. Our finding, that the use of these methods is also associated with higher medicine availability, is consistent with a recent study in Ethiopia, which also showed a link between availability and these inventory management principles.^39^ In Indonesia, the use of FEFO principles should be combined with the use of a digital inventory management system, named LPLPO (Laporan Pemakaian dan Lembar Permintaan Obat), which is also associated with higher medicine availability. Together, FEFO principles and LPLPO inventory management can help prevent shortages and waste. To truly benefit from these systems, the health sector needs to address challenges such as manual reporting, logistics issues and limited digital integration.

A key component of effective pharmaceutical services is promoting the rational use of medicines. While our study did not assess actual dispensing practices, our analysis shows that reporting on rational medicine use is associated with higher medicine availability. This finding may reflect the clustering of various aspects of good pharmaceutical practice at the local level. These patterns need to be explored further in qualitative studies and could perhaps be a starting point for developing interventions aimed at improving pharmaceutical practices.^40^

Our study found that essential medicine availability in PHCs was closely tied to the availability at DHOs. Districts with more proactive DHOs that employed a variety of purchasing channels generally had better medicine availability. In contrast, DHOs relying on a single procurement strategy often faced shortages, leaving health facilities and communities without essential medicines. Additionally, districts experiencing frequent payment delays tended to have lower medicine availability, likely due to reduced supplier willingness to deliver medicines. These findings highlight the need to strengthen the capacity of DHOs in managing pharmaceutical supply chains. Special attention should be given to improving supply chains in remote districts and PHCs, which are particularly prone to shortages.^26^ Pooling procurement for these areas could be an effective strategy, as increasing order volumes may make distribution more attractive to suppliers.^41 42^

Our analysis reveals that medicine availability is shaped not only by the district health office but also by local clustering patterns among PHCs. This local clustering of medicine availability was significant. We found clusters of PHCs in Java Island with low medicine availability, and clusters in relatively poor parts of East Nusa Tenggara, Sumatra and Maluku provinces with high medicine availability. This clustering, observed within a few kilometres, appears to be driven by two key factors. First, nearby PHCs may collaborate by sharing stock during shortages or referring patients to other facilities for specific health programmes.^26^ Second, medicine distribution may be influenced by established supply routes, contributing to the observed clustering. These findings highlight the significance of local dynamics in determining medicine availability, suggesting that interventions aimed at improving access should consider localised strategies. Further research is needed to evaluate the extent of stock-sharing practices and identify ways to optimise them for enhanced medicine availability.

Our findings highlight the need to consider local systems and contextual factors when aiming to improve access to medicines. This observation is in line with a recent study in Afghanistan, which used the same LOPHAS framework and methodology, revealing that variations in medicine availability were less affected by the security situation and more by the type of organisation managing healthcare, with non-governmental organisations (NGOs) generally performing better than local government entities.^6^

### Policy implications

Given Indonesia’s vast size and regional diversity, it is worth questioning whether a single national essential medicines list is suitable for all districts across the archipelago. Currently, Indonesians enrolled in the National Health Insurance Scheme are entitled to free access to nearly 300 medicines through their PHCs. Our results show that even among the 50 most commonly used medicines, a significant number are frequently unavailable. This inconsistency generates uncertainty for both patients and healthcare providers, undermines trust in the public healthcare system and often compels patients to seek medicines from alternative sources, increasing their risk of exposure to costly, expired, substandard or even falsified medicines.^43 44^

One potential strategy to improve access to medicines is to streamline the list of promised medicines, focusing on a smaller, more manageable selection that can be consistently supplied. By narrowing the scope, health systems can allocate resources more effectively, ensuring reliable availability of essential medicines rather than overpromising and underdelivering. In parallel, increasing the number of PHCs staffed with qualified pharmacists can directly enhance medicine availability, as pharmacists play a critical role in inventory management, procurement and rational dispensing. Additionally, providing up-to-date, transparent information on medicine availability at each PHC could enhance patient trust and enable healthcare providers to make more informed prescribing decisions. Together, these strategies not only help manage patient expectations and reduce unnecessary stockouts but also strengthen the credibility, efficiency and overall performance of the healthcare system.

Additionally, a more localised approach to essential medicines lists—tailored at the provincial or district level—could better reflect regional healthcare needs and practical conditions.^45^ A similar model, the Municipal Essential Medicine Lists (MEMLs) has been implemented in Brazil and is used effectively to assess local PHCs and reduce stock-outs.^46^ Adopting a comparable system in Indonesia may help align procurement with local needs, provide greater clarity to patients regarding medicine availability and enable healthcare providers to better meet expectations while enhancing accountability within the system.

### Strengths and Limitations

This is the first study to use nationwide data from all districts, DHOs and PHCs in Indonesia, incorporating a wide range of health system and contextual indicators. By combining these datasets, we were able to conduct comprehensive multilevel and spatial analyses of medicine availability in one of the world’s largest and most diverse nations. A key limitation is that our data reflect availability only in public facilities and do not capture the extent to which local communities can access these medicines. Additionally, measurements were taken at a single point in time, so temporal fluctuations in availability are not captured, although the large sample size reduces the likelihood of major deviations. Importantly, this study examines associations between variables and medicine availability, which does not establish causality. As a result, observed patterns should be interpreted as indicative of potential relationships rather than definitive causal effects. Future research using longitudinal or quasi-experimental designs could provide stronger evidence of causal links and guide targeted interventions to improve medicine availability.

### Conclusion

Overall, this study demonstrates that the availability of essential medicines in Indonesian PHCs varies widely across districts and is closely linked to the functionality of local pharmaceutical systems. Ensuring consistent medicine availability requires a multifaceted approach that addresses both facility-level and district-level factors. Our findings underscore the importance of investing in the physical and human resources of PHCs, particularly by ensuring the presence of trained pharmacists. Furthermore, the application of sound inventory management practices and strong coordination between PHCs and district health offices is critical for optimising medicine distribution. While locally specific initiatives to strengthen PHCs are important, targeted interventions that build the capacity of DHOs to manage pharmaceutical supply chains are equally vital. Strengthening these local pharmaceutical systems will be essential to achieving a reliable, equitable supply of essential medicines across Indonesia’s diverse regions.

## Supplementary material

10.1136/bmjgh-2025-019616 online supplemental file 1

10.1136/bmjgh-2025-019616 online supplemental file 2

10.1136/bmjgh-2025-019616 online supplemental file 3

## Data Availability

Data are available upon reasonable request.
