# Supplementary material for: Local systems, local solutions: which factors drive essential medicine availability in public health facilities across Indonesia?
Source: BMJ Glob Health. 2026 Feb 6;11(2):e019616. doi: 10.1136/bmjgh-2025-019616 (PMC12887493; doi:10.1136/bmjgh-2025-019616)
Supplement: online supplemental file 1 [file bmjgh-11-2-s001.docx]

**Author reflexibility statement**

Title: **Local systems, local solutions: which factors driving essential medicine availability in public health facilities across Indonesia**

1. How does this study address local research and policy priorities?

The Indonesian government strives to achieve UHC, which includes providing free access to essential medicine in its public facilities. While multiple factors are associated with the performance of local pharmaceutical systems, it remains unclear which factors are most influential in ensuring access to medicines including medicine provision. This study is in line with a key priority of the Ministry of Health, which is to ensure that essential medicines are available in all PHCs across the country.

1. How were local researchers involved in study design?

The health facility survey (Rifaskes 2019), which forms the core of this study, was primarily designed by Indonesian researchers, who designed the survey, organized the pilot and the data collections in the PHCs. Local researchers also played a key role in designing the analysis for the manuscript.

1. How has funding been used to support the local research team?

The Indonesian government funded these works, covering operational costs for Rifaskes 2019 and manuscript development. This funding support aims to enhance the research capacity of the local team.

1. How are research staff who conducted data collection acknowledged?

Two Indonesian scholars from Balitbankes, who led the survey are co-authors. Three other Indonesian scholars who worked with the survey team and analysis are also co-authors. There were 2056 data collectors involved, who are thanked in the acknowledgements section.

1. Do all members of the research partnership have access to study data?

All members of the research partnership who worked on the study survey had access to the dataset.

1. How was data used to develop analytical skills within the partnership?

The first author is a PhD candidate from Indonesia. The PhD work included development of analytical skills for this PhD candidate, and other scholars involved.

1. How have research partners collaborated in interpreting study data?

All research partners were involved in interpreting the study results, during multiple online and offline meeting, exchanges and discussion sessions.

1. How were research partners supported to develop writing skills?

The research partners, from different institutions in Indonesia and Netherlands, closely collaborated in the writing of the manuscript, and learned from each other through feedback, critical reading and by providing suggestions, which helped to strengthen writing skills.

1. How will research products be shared to address local needs?

After submitting this manuscript, the team plans to disseminate the research findings to the Indonesian Ministry of Health and other key stakeholders working to improve access to medicines in Indonesia. We are drafting a policy brief, short video and social media strategy.

1. How is the leadership, contribution and ownership of this work by LMIC researchers recognised within the authorship?

Five out of eight authors are from Indonesia, including the first, second and third author.

1. How have early career researchers across the partnership been included within the authorship team?

There are four early career researchers included in the authorship team, including three PhD candidates and a MSc student.

1. How has gender balance been addressed within the authorship?

Three of the eight authors identify as female. Five identify as male.

1. How has the project contributed to training of LMIC researchers?

As stated previously, there are four early career researchers included in the study team, including three PhD candidates and a graduate student. All involved researchers learned from contributing to this project, by gaining experience, mentoring and peer-feedback and formal trainings.

1. How has the project contributed to improvements in local infrastructure?

The Indonesian led health facility survey was designed to produce data for improving and strengthening multiple national policies in Indonesia, such as strengthening the district health system. The findings can be used by Indonesian policymakers to improve system performance, including better access to medicines in public facilities across the country.

1. What safeguarding procedures were used to protect local study participants and researchers?

The survey has received Ethical approval from the Health Research Ethics Committee, National Institute of Health Research and Development (HREC – NIHRD) with reference number LB.02.01/2/KE.011/2019. Before the data collection began, participants received an explanation about the survey aims and participant rights. Data are stored at password protected devices and server facilities at all times.
