# Supplementary material for: Local systems, local solutions: which factors drive essential medicine availability in public health facilities across Indonesia?
Source: BMJ Glob Health. 2026 Feb 6;11(2):e019616. doi: 10.1136/bmjgh-2025-019616 (PMC12887493; doi:10.1136/bmjgh-2025-019616)
Supplement: online supplemental file 2 [file bmjgh-11-2-s002.docx]

**Supplemental Table and Figure**

Supplemental Table 1. The list of Covariates in this study

|  | Covariate names | Covariates description |
| --- | --- | --- |
|  | **PHC Level** |  |
|  | ***Local Pharmaceutical Systems*** |  |
|  | *Accessibility, financial and demographical determinants* | |
| 1 | PHC type | The type of PHCs based on MoH classification in 2019:   - Remote/very - Rural - Urban |
| 2 | District capital point | Distance between the PHC with the central point of its district in Kilometres (KM^2^) |
| 3 | Provincial capital point | Distance between the PHC with the central point of its provincial in KM^2^ |
| 4 | Nearest PHCs point | Distance between the PHC with the central point of its nearest neighbours in KM^2^ |
|  | *Managing Human &Physical Resources* | |
| 5 | The availability of pharmacists | The PHC has at least one staff having educational background and certificated as pharmacist |
| 6 | The availability of Medicine Shelves | The PHC has at one medicine shelve available in the PHC pharmaceutical room. |
| 7 | Perceived of Sufficient number of Shelves | The PHC has enough numbers of medicine shelves to store their medicines in its pharmaceutical room. |
| 8 | The availability of Ventilation | The PHC has at least one of ventilation or air circulation and preventing the accumulation of excess moisture. |
| 9 | Lighting availability | The PHC has at least one of lighting in its pharmaceutical room. |
| 10 | Pharmaceutical room | The PHC has a room for storing its medical and pharmaceutical product |
| 11 | Distribution guidelines | The availability of the guidelines of Internal medicine and medical product distribution |
| 12 | Service guidelines | The availability of the guidelines of Medicine and medical product services |
| 13 | Concoction guidelines | The availability of the guidelines of Preparing and dispensing medicine concoction prescription |
| 14 | Dry syrup guidelines | The availability of the guidelines of Preparing and dispensing dry syrup |
| 15 | Inpatient services | The PHC is serving inpatient services, which allow patient to stay in a hospital or other care facility overnight. |
| 16 | Been accredited | Has been accredited by the national accreditation agency |
|  | *Financing* |  |
| 17 | National fund | Receiving ‎a funding scheme from the central government |
| 18 | District fund | Receiving a fund from the district government |
| 19 | Patient retribution | Receiving retribution from patients |
| 20 | Capitation fund | Receiving Capitation fund from the National Health Insurance (BPJS Kesehatan) |
| 21 | Non-capitation fund | Receiving non-capitation fund from the BPJS Kesehatan |
| 22 | Financial management | Implementing independent financial management which allows PHCs to determine how they will spend their own expenditure. |
|  | *Monitoring Performance* |  |
| 23 | SIMPUS availability | Having an internal management information system (SIMPUS) to administer all works in the PHC. |
| 24 | Electronic SIMPUS | Having the SIMPUS in electronic type |
| 25 | Online SIMPUS | Having the SIMPUS online presence |
|  | *Managing Product Supply* |  |
| 27 | LPLPO availability | The availability of Medicine use and ordering monthly report called LPLPO |
| 28 | Completed LPLPO | All the LPLPO reports in 2018 are found from January to December |
| 29 | DRP 18 months | Implementing Drug Requirement Plan (DRP) planning for 18 months period |
| 30 | Develop DRP | Able to develop the PHC DRP. |
| 31 | Perform independent procurement | PHCS is able to procure their medicines independent |
| 32 | Fully received from DHO | All medicines are from DHO procurements |
| 33 | Independent procurements | All medicines are from independent procurements |
| 34 | Combining DHO & PHC | All medicines are from DHO & PHC |
| 35 | Achieved target | Achieving the target of the needed medicine volume based on its DRP. (at least 80% of the DRP). |
| 36 | Use capitation fund | Able to use capitation fund to procure medicine and other pharmaceutical products, if the DHO was not able to provide the needed medicines with an approval letter from its DHO. |
| 37 | Use capitation independently | Independently procure medicines using capitation fund without any approval from the DHO., |
| 38 | Purchase via e-catalogue | Perform a medicine procurement using the capitation fund via e-purchasing scheme |
| 39 | Purchase via direct purchasing | Procuring using capitation fund through direct purchasing |
| 40 | Note 2018 | The PHC has its note/report for medicine stock in and out in 2018 in its warehouse. |
| 41 | FEFO Method | Use method First Expired First Out to maintain the medicine stocks based on its expiry date to prevent the waste. |
| 42 | Rational use report | The PHC evaluate the medicine use appropriateness to patients based on the volume and reporting the rational use to its DHO. |
| 43 | Implementing PRB program | The PHC is implementing PRB program, which serves patients with non-communicable diseases (Hypertension, Cardiovascular diseases, and Diabetes Meletus) needs of medications. |
|  | ***District Level*** |  |
|  | *Accessibility, financial and demographical determinants* | |
| 44 | Type of district | Type of district based on the Ministry of Home Affair in 2019:   - Rural district - Urban district |
| 45 | Percentile group population | Percentile group of district population based on total number of populations residing in a district from Q1 (Small number of the population) to Q5 (high number of the population |
| 46 | District Percentage PBI | Percentage of Number of JKN Subsided Participants (People with limited income population) in 2019 in its district |
| 47 | District health expenditure | The percentage of health Expenditure compared to total district government expenditure |
| 48 | District Fiscal Capacity | District financial capacity based on its revenues in 2018 per capita. |
| 49 | District Accessibility alternative | The score of accessibility for any other dispensing point at the district level, which reflects to option to find another dispensing point. |
| 50 | Total district expenditure | The total of district expenditure in 2019 to fund all government activities |
| 51 | Separated_ Island | The PHC is located in a separated island from the capital of the province |
|  | *Local Pharmaceutical system* |  |
|  | *Managing Human & Physical Resources* |  |
| 52 | Pharmacist at warehouse | At least one pharmacist work to manage medicines in the district medicine warehouse |
| 53 | The PIC of the Warehouse is a pharmacist | Having a pharmacist as the Person in Charge at the district warehouse |
| 54 | Ideal pharmacist team in warehouses | Having three pharmacists as ideal composition for pharmaceutical team in district warehouses, one is responsible for managing and distributing all PHC needs, one is managing medicine procurements with medicine suppliers, and other is taking responsible for monitoring and managing medicines from the MoH. |
|  | *Financial Capacity* |  |
| 53 | Using National fund | The DHO received and used the national fund to procure their medicines |
| 54 | Using Provincial Fund at DHOs | The DHO received and used the provincial fund to procure their medicines |
| 55 | Using district Fund | The DHO received and used the district fund to procure their medicines |
| 56 | Using Capitation Fund | The DHO received and used the capitation fund from its PHCs to procure their medicines |
|  | *Managing Product Supply* |  |
| 57 | Best lead time | The lead time refers to the total amount of time it takes to complete a process from submitting a DHO’s medication application to the DHO receives the targeted medicines. The best lead-time was calculated as how fast it takes, which are:   - Within 14 days - 15 – 30 days - 31 – 90 days - More than 90 days. |
| 58 | DHO medicine quantification | The DHO has developed its own medicine quantification plan to procure medications |
| 59 | DHO E-catalogue | The DHO is able to procure medicines via the e-catalogue platform |
| 60 | DHO Local Auction | The DHO is able to procure medicines in a local auction scheme after failing to procure the medicines from the e-catalogue platform |
| 61 | DHO Direct purchasing | The DHO is able to procure medicines in a direct purchasing scheme after failing to procure the medicines from the e-catalogue platform |
| 62 | DHO Implemented 2 years of expiry date | The DHO requires that some procured medicines should have two years of date expiration as mandated by the national regulation in medicine management. |
| 63 | DHO all 2 years | The DHO requires that all procured medicines should have two years of date expiration as mandated by the national regulation in medicine management. |
|  | *Medicine availability* |  |
| 64 | DHO medicine availability | The rate of availability of 50 selected medicines at the district warehouse. |
|  | ***Provincial Level*** |  |
| 65 | Province Population | Number of populations in the province, which is grouped to five categories (Q1 (Small number of the population) to Q5 (high number of the population) |
| 66 | Province Percentage PBI | Percentage of Number of JKN Subsided Participants (People with limited income population) in 2019 in its province |
| 67 | Provincial health expenditure | The percentage of health Expenditure compared to total government expenditure at provincial level |
| 68 | Provincial fiscal capacity | Province financial capacity based on its revenues in 2018 per capita. |
| 69 | Province Accessibility alternative | The score of accessibility for any other dispensing point at the district level, which reflects to option to find another dispensing point |
| 70 | Regions | Group of Region based on the similar characteristics:   - Eastern Indonesia - Sumatera - Borneo, West Nusa Tenggara, Sulawesi - Java and Bali |

Supplemental Table 2. The average of medicine availability based on geographical accessibility and fiscal capacity, district health office and primary health centre characteristics.

|  | Median | Interquartile range |
| --- | --- | --- |
| PHC Level |  |  |
| *Accessibility, financial and demographical determinants* |  |  |
| Distance between PHC and its district capital point | 13,90 | 19,08 |
| Distance between PHC and its provincial capital point | 86,2 | 105,31 |
| Distance between PHC and the nearest PHC | 4,14 | 5,67 |
| District Level |  |  |
| *Accessibility, financial and demographical determinants* |  |  |
| Percentage of health expenditure from total expenditure | 14,82 | 4,68 |
| Percentage of Subsidised JKN participants | 69,60 | 26,20 |
| Accessibility score to other dispensing point | 95,00 | 22.50 |
| Provincial level |  |  |
| *Accessibility, financial and demographical determinants* |  |  |
| Percentage of health expenditure from total expenditure | 8,70 | 6,70 |
|  | **Mean** | **Standard deviation** |
| District Level |  |  |
| *Local Pharmaceutical systems* |  |  |
| *Medicine availability at the DHO level* |  |  |
| The availability of 50 medicines | 72.9 | 19.54 |


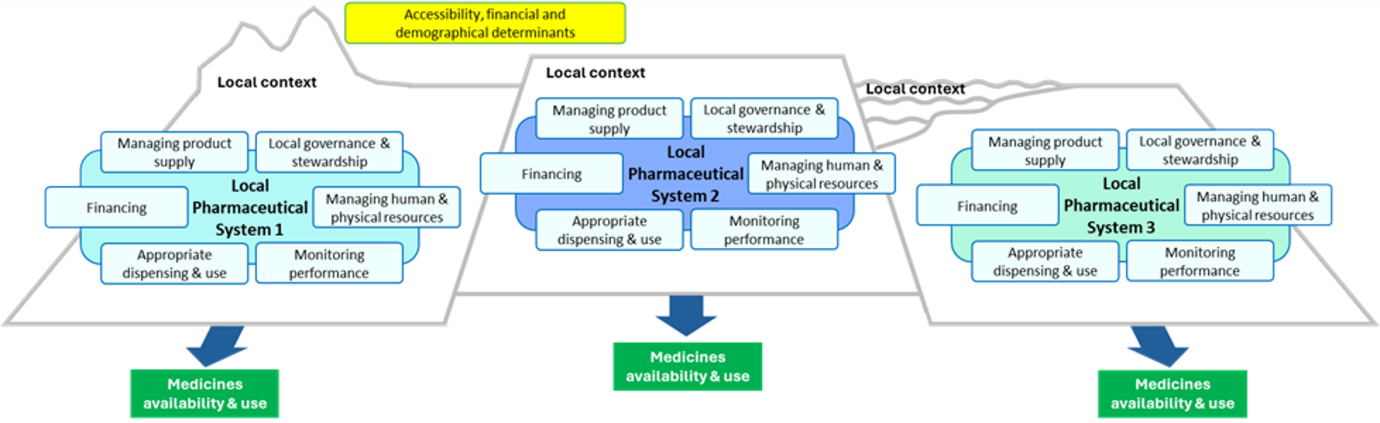
 Supplemental Figure 1. Key functions of local pharmaceutical system


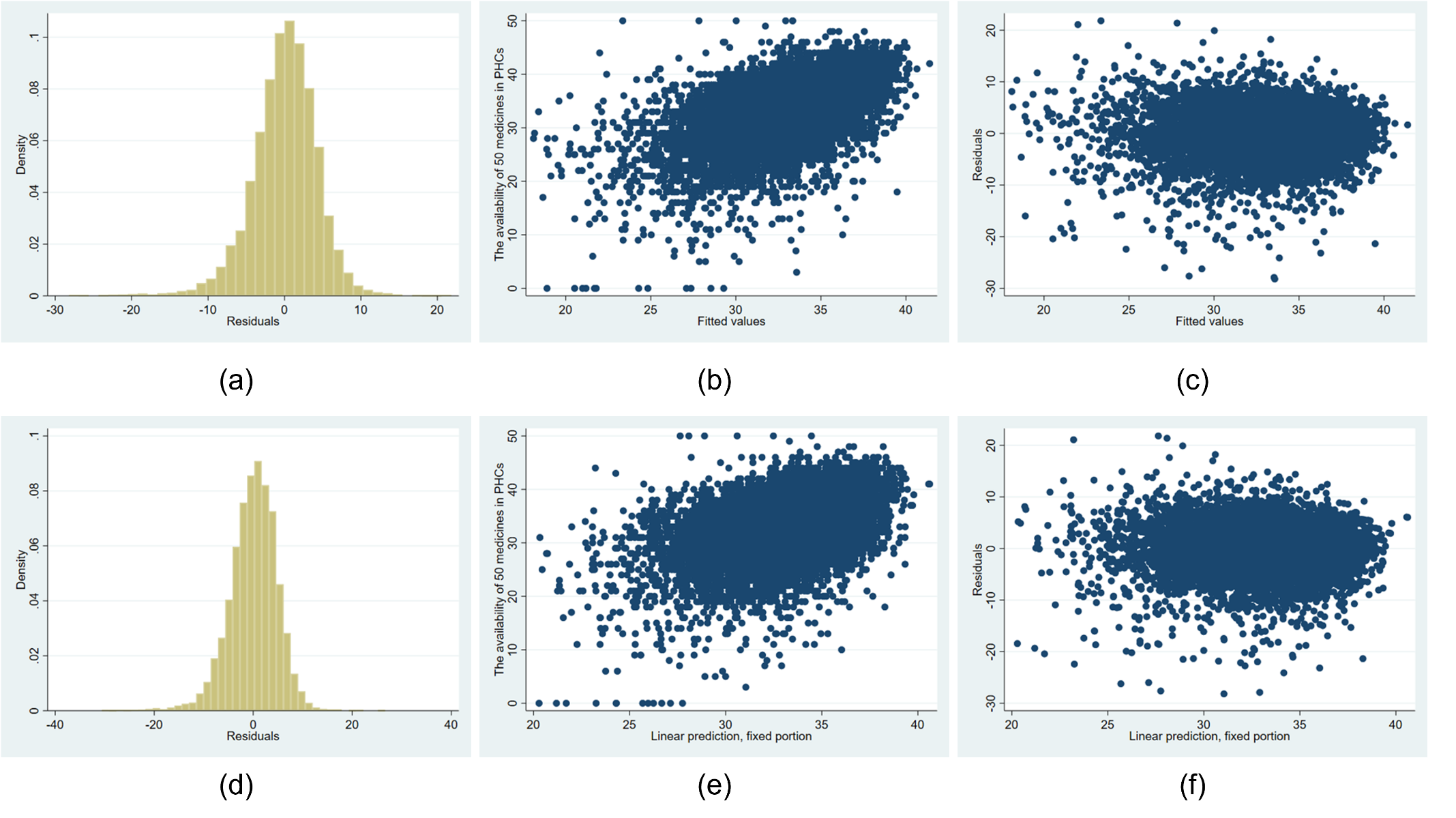


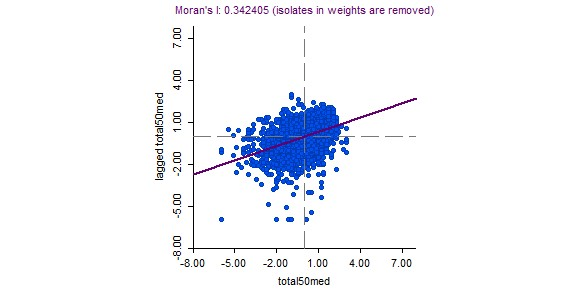


Supplemental Figure 3. A local indicators of spatial association analysis. X-axis normalized availability of medicines in health facility, Y-axis: normalized availability of medicines of neighboring facilities (10 km inverse distance weights).


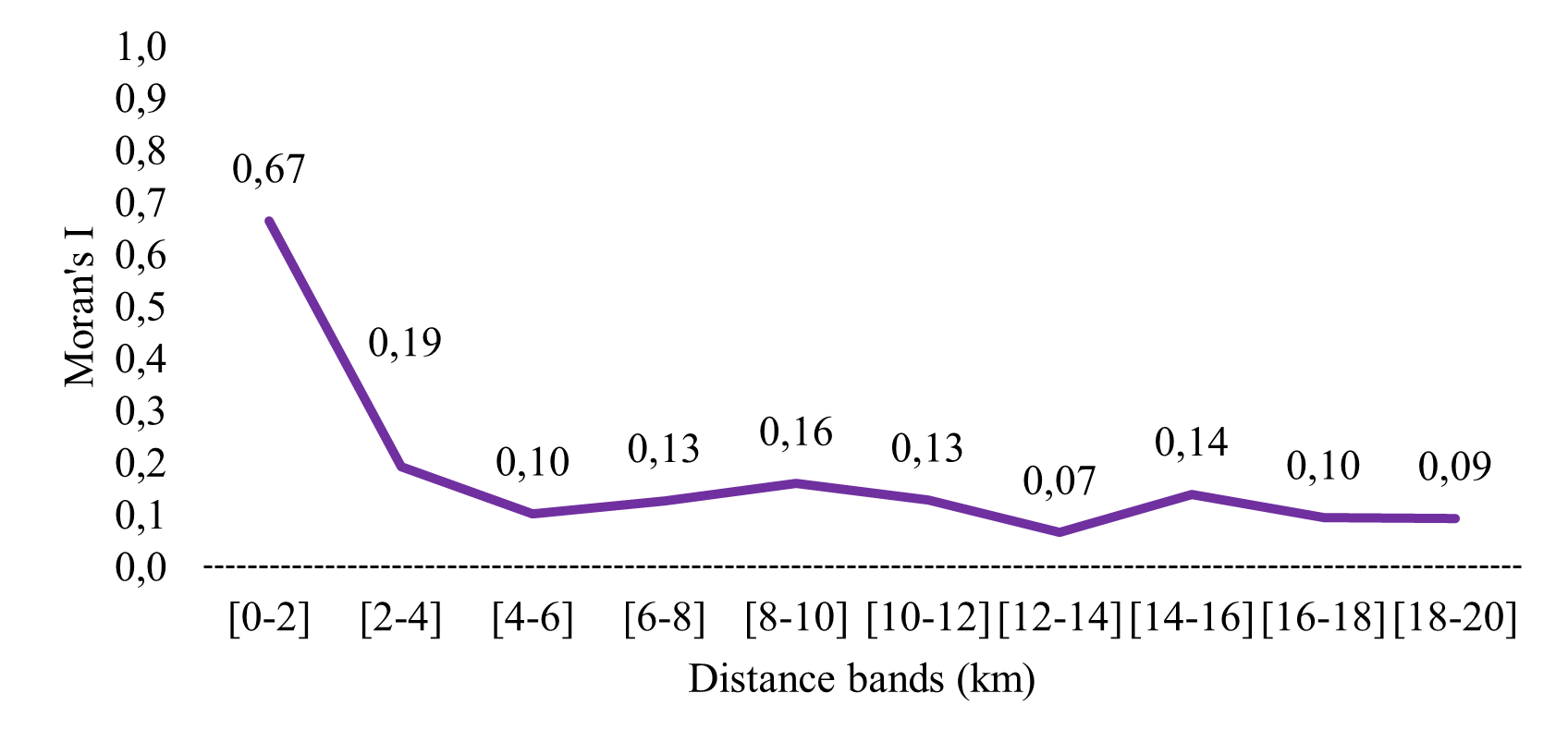


Supplemental Figure 4. Spatial autocorrelation of availability of medications at PHC level within a two-kilometre distance
